# The Gross National Happiness Framework and the Health System Response to the COVID-19 Pandemic in Bhutan

**DOI:** 10.4269/ajtmh.20-1416

**Published:** 2020-12-22

**Authors:** Thinley Dorji

**Affiliations:** Jigme Dorji Wangchuck National Referral Hospital, Thimphu, Bhutan;; Kidu Mobile Medical Unit, His Majesty’s People’s Project, Thimphu, Bhutan

## Abstract

Bhutan is a lower-middle–income country with limited tertiary-care health infrastructure and shortage of human resources. The country’s response to the COVID-19 pandemic is guided by the principle of Gross National Happiness (GNH), which prioritizes the well-being and happiness of people over conventional socioeconomic indicators. The king’s leadership and government’s decisions based on public health science helped in the control of the pandemic and reduce economic losses. The government implemented some unique and unconventional public health measures such as facility quarantine for those with high-risk exposure, an increase in quarantine period to 21 days, free testing and treatment, and population-based screening tests. Early and extensive contact tracing, extensive testing, effective communications, zoned travel restrictions, and adoption of physical distancing and hygiene measures limited COVID-19 transmissions within the country. Community participation from voluntary groups and civil society organizations helped deliver non-health services while hospitals provided uninterrupted routine health services through its primary healthcare network. All COVID-19 cases were treated in hospitals, and the country has had zero reported COVID-19 deaths. This article describes how the concept of GNH provided the framework for the government to respond to this pandemic.

## INTRODUCTION

**Figure 1.**
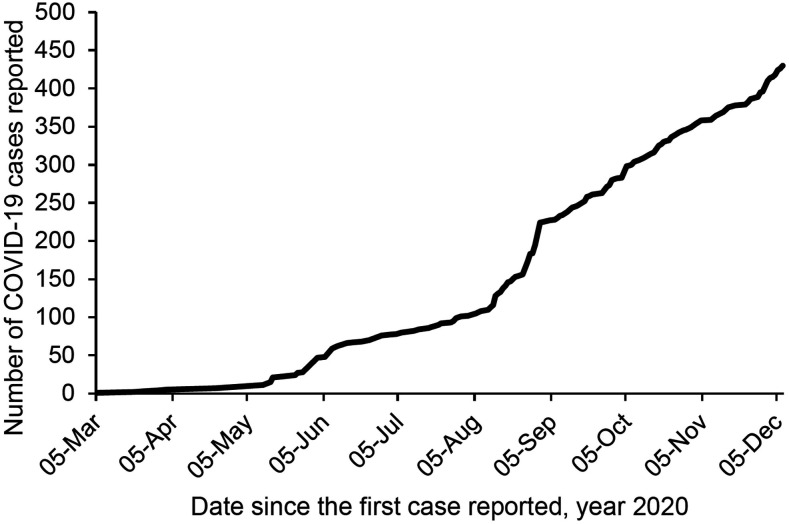
The number of COVID-19 cases in Bhutan from March to December 7, 2020 reported by the Ministry of Health, Royal Government of Bhutan.

Bhutan is a small Himalayan country that partly shares a porous border with the northeastern part of India, the latter with 10 million cases of COVID-19 with grossly underreported deaths.^1^ As of December 7, 2020, 430 cases of COVID-19 were detected in Bhutan and has had zero reported deaths (Figure 1). The country with a limited number of human resources and tertiary-care infrastructure aims to limit the local spread and prevent COVID-19–related deaths as its primary goal. The country’s response to the impacts of the COVID-19 pandemic is guided by the framework of Gross National Happiness (GNH), which is an overall policy guide for Bhutan.

Gross National Happiness was introduced in the 1970s to guide development toward building conditions that enhance people’s happiness and well-being.^2^ The conceptual framework of GNH is based on four pillars: sustainable and equitable socioeconomic development, environmental conservation, preservation and promotion of culture, and good governance; nine domains: psychological well-being, health, time use and balance, education, cultural diversity and resilience, good governance, community vitality, ecological diversity and resilience, and living standards; and 33 indicators contributing to the GNH index. Table 1 describes the domains and indicators of GNH measurement.^2^ This article discusses how the country’s health policy and system responded to the challenges and impacts of the COVID-19 pandemic in Bhutan, and is interpreted within the GNH domains of health, psychological well-being, good governance, and community vitality.

**Table 1:** The nine domains and 33 indicators that measure Gross National Happiness in Bhutan^2^

Domain	Indicators
Psychological well-being	Life satisfaction
	Positive emotions
	Negative emotions
	Spirituality
Health	Self-reported health
	Healthy days
	Disability
	Mental health
Time use	Work
	Sleep
Education	Literacy
	Schooling
	Knowledge
	Value
Cultural diversity and resilience	*Zorig Chusum* (artistic skills)
	Cultural participation
	Speak native language
	*Driglam namzha* (a way of harmony)
Good governance	Political participation
	Services
	Governance performance
	Fundamental rights
Community vitality	Donation (time and money)
	Safety
	Community relationship
	Family
Ecological diversity and resilience	Wildlife damage
	Urban issues
	Responsibility toward environment
	Ecological issues
Living standards	Per capita income
	Assets
	Housing

## GROSS NATIONAL HAPPINESS DOMAIN: HEALTH

### Infrastructure.

Bhutan’s population of 0.7 million is scattered across mountainous terrain in 20 districts with relatively higher population located in the capital city Thimphu and in the southern districts that share a porous border with India. As COVID-19 spread around the world, the health system in Bhutan was evaluated and simulated against mathematical models of disease spread even before the first case was detected in the country. In 2020, there were 48 hospitals, 186 primary health centers, and three municipality health centers that covered more than 95% of the population within 3 hours of travel.^3^

Every hospital established flu clinics that were standalone and separate from the main hospital infrastructure to screen for influenza-like illness and COVID-19, and designated separate facilities to isolate COVID-19 cases. Apart from the Royal Centre for Disease Control in Thimphu, reverse transcription (RT)-PCR testing facilities were newly established in four other geographically strategic locations (regional referral hospitals in Monggar and Gelegphu, and general hospitals in Phuentsholing and Dewathang) to provide timely access to tests. The existing intensive care facilities at the three tertiary hospitals were strengthened with additional equipment and human resource. In Phuentsholing, the second most populous town and an economic hub in the south along the borders with India, new intensive care setup and RT-PCR testing facilities were established. COVID-19 antibody and antigen tests are available at all levels of hospitals.

### Human resources.

The country has limited doctors, nurses, and technologists, and no infectious disease specialists, virologists, or immunologists. In 2020, there were 376 doctors and 1,364 nurses with a ratio of 5.6 doctors and 18.4 nurses per 10,000 population.^3^ After the detection of the first COVID-19 case in March 2020,^4^ human resources were reappropriated according to the population at risk. Additional doctors, nurses, and laboratory technologists were deployed in high-population areas such as Thimphu, Paro, and Phuentsholing. Fifty doctors undergoing postgraduate training outside the country (13.3% of total registered in the country) were recalled, and 25 newly graduated doctors, nurses, and technologists were recruited through a fast-tracked process.^5^ Given the lack of adequate doctors and nurses trained in critical care, 77 doctors and 255 nurses were trained in the management of intensive care units.^6^

The Royal Centre of Disease Control engaged students from the Faculty of Nursing and Public Health, Khesar Gyalpo University of Medical Sciences of Bhutan in running the RT-PCR tests. Volunteers from the *De Suung* Guardians of Peace were engaged in monitoring of persons in quarantine facilities and enforcing physical distancing of crowds in hospitals and public places.

### Health services: Routine and COVID-19 services.

The country provided uninterrupted routine services such as immunization, maternal and child health services, and treatment of other chronic conditions such as diabetes, hypertension, tuberculosis, and HIV, demonstrating its commitment to primary health care. Healthcare service delivery and routine outpatient services were redesigned to ensure continued accessibility while preventing cross-contamination with potential COVID-19 patients. To limit hospital visits, 54 flu clinics were established in physically separate locations that conducted passive surveillance and COVID-19 testing for all influenza-like illness, whereas hospitals initiated mobile delivery of services and adoption of innovative measures such as designating drop-in places for delivery of medicines including contraceptives.^7^ After an increasing number of COVID-19 cases were detected in several districts in the south, all inpatients and attendants were tested for COVID-19 antigen and RT-PCR before admission in hospitals.

COVID-19 cases are treated at four designated centers to concentrate the cases and rationalize the use of staff and personal protective equipment: the Jigme Dorji Wangchuck National Referral Hospital in Thimphu, Phuentsholing Hospital, Central Regional Referral Hospital in Gelegphu, and Eastern Regional Referral Hospital in Monggar (Table 2). As of November 20, 2020, 491 health staff were deployed for 304 COVID-19 patients, with a health staff to patient ratio of 1.6:1.^6^ According to the National COVID-19 Treatment Guideline, all COVID-19 cases including those asymptomatic are admitted and treated in hospitals followed by 2 weeks of facility de-isolation and require at least two negative RT-PCR tests before discharge from de-isolation.^8^

**Table 2:** Number of cases managed at the four COVID-19–designated hospitals in Bhutan as of November 20, 2020

COVID-19 centers in Bhutan	COVID-19 patients	Staff deployed*
	*n* (%)	*n* (%)
Jigme Dorji Wangchuck National Referral Hospital, Thimphu	149 (49.0)	277 (56.4)
Phuentsholing General Hospital, Phuentsholing	144 (47.4)	124 (25.3)
Central Regional Referral Hospital, Gelegphu	6 (2.0)	58 (11.8)
Eastern Regional Referral Hospital, Monggar	5 (1.6)	32 (6.5)
Total	304 (–)	491 (–)

* Staff deployed, inclusive of doctors, nurses, and supporting staff.

### Health finances and resources.

The free healthcare services are funded by the government with a budget allocation of 3.6–6.9% of gross domestic product per year, and essential medicines and vaccines are procured through the Bhutan Health Trust Fund.^9^ For the fiscal year 2020–2021, the government allocated Nu 6,438 million (≈United States dollar [USD] 87 million) for the health sector, an increase of 11% compared with the previous year.^10^

A special COVID-19 fund was created with government allocation of budget and donations from citizens and international organizations.^11^ All COVID-19 testing and treatment are provided free of cost by the government, and there is no out of pocket expenditure; COVID-19 vaccines will also be provided free of cost to all citizens. The government procured COVID-19 test kits and reagents besides receiving donations from the government of India and international organizations.^12^ All health workers are insured by the government for COVID-19–related death.

### COVID-19–related public health actions and surveillance.

The government’s strategy for preventing the spread of COVID-19 in the country includes four key areas of intervention: testing, tracing, treating, and behavior change. As of September 2020, the testing rate stood at 146,852 tests per million population, 45th in global ranking with 59,543 RT-PCR tests and 59,543 rapid diagnostic tests conducted.

The government adopted some unique public health measures that have proved beneficial in the local context. Those Bhutanese who were repatriated to the country were quarantined in government-sponsored facilities for a duration of 21 days in contrast to the World Health Organization recommendation of 14 days.^13^ This decision incurred additional costs for the government (Nu 248.28 million, ≈USD 3.4 million, from March to September 2020) but led to the detection of several positive cases in the third week of facility quarantine, preventing exposures in the community. The completion of quarantine period was coupled with RT-PCR tests for those with symptoms and antibody testing for others. From March till August 2020, all cases were detected within quarantine centers, allowing others to live normal social lives until a nationwide lockdown was imposed in August after the detection of the first case outside facility quarantine centers.

In addition to the existing National Early Warning Alert and Response Surveillance system, the Royal Centre for Disease Control established a COVID-19–integrated influenza surveillance system. Under this system, random samples of mobile population in high-population towns are randomly tested with COVID-19 antibody tests. All severe acute respiratory illness and influenza-like illness are tested for COVID-19. The entire population was given influenza immunization in October–December 2020.

The country also adopted rigorous and early contact tracing with 90 contacts traced overnight in relation to the first case in March 2020 and 97 cases traced overnight in relation to the first case of COVID-19 outside quarantine facility in Gelegphu in August 2020.^14^ Vigilant monitoring of disease transmission and active surveillance with random sampling tests were performed in Thimphu and Phuentsholing. All healthcare workers in high-risk hospitals and those working in high-risk sectors such as border patrolling and personnel at the point of entries along the international border are tested for COVID-19 on regular intervals.

When cases were detected outside quarantine centers in Gelegphu and Phuentsholing, the government initiated a national lockdown that lasted 21 days. The cluster of COVID-19 cases was reported among manual workers at the mini-dry port that served as the point of entry of goods from India into Bhutan. During this period, the government initiated population testing of all persons older than 10 years in Phuentsholing, totaling more than 31,486 RT-PCR tests. In addition, 7,484 people who had traveled out of Phuentsholing 2 weeks before the initiation of lockdown were also tested. This was followed by testing of people residing along the Phuentsholing–Thimphu national highway, the route that sees the highest traffic out of Phuentsholing. This population-based screening led to the detection of a total of 111 cases (49% of total on September 1, 2020) in several self-contained sporadic clusters of outbreak.^15^ As of September 2020, the government also tested 4,039 individuals from foreign institutions (embassies, donor agency offices, etc) in the country. After the detection of another case outside quarantine facility in Samdrup Jongkhar town, a district in the east bordering India, whole population testing was adopted in the district.

### Communications during COVID-19.

The government had a comprehensive communication strategy based on the theme “our *gyenkhu*—our responsibility.” The prime minister, ministers, and key representatives of organizations were frequently on television, radio, and social media to communicate both real and potential risks and to take questions from the media. The effectiveness of these communications was seen with compliance to social distancing measures, use of face mask, handwashing, use of Druk Trace mobile phone application, and minimal quarantine and lockdown violations. Media outlets and social media influencers carried health messages in multiple languages and dialects and addressed fake news and unauthenticated information.

## GROSS NATIONAL HAPPINESS DOMAIN: PSYCHOLOGICAL WELL-BEING

The pandemic has caused disruption in socioeconomic activities, education, and other planned development activities. In addition, the news of COVID-19–related mortality had an immense impact on psychological well-being. A national mental health response team and sub-teams across the countries provided psychological first aid and counseling.^16^ As the impact of this pandemic remained protracted, these teams dealt with an increasing number of mental health–related issues.

## GROSS NATIONAL HAPPINESS DOMAIN: GOOD GOVERNANCE

Bhutan has a Democratic Constitutional Monarchy. The king has played a central role in leading the country’s response. King Jigme Khesar Namgyal Wangchuck emphasized in a televised national address that “the country must rise above all other considerations and continue to provide substantive, timely, and inclusive support to sustain public confidence and build resilience in these challenging times.” The king announced a National Resilience Fund (Nu 30 billion, ≈USD 405 million) and monetary and fiscal measures such as waiver of loan interests, and financial support to those rendered jobless through the *Druk Gyalpo’s Relief Kidu.*^17^ The king constructed shelters and migrated 5,000 Bhutanese to Phuentsholing who were living in neighboring Indian town of Jaigaon before the latter was hit by increasing cases of COVID-19. The king also announced the closure of Bhutan’s international border from March 23, 2020 onward in the wake of travel-related COVID-19 cases.

The democratic government elected in 2018 has a heavy representation of health sciences in the 11-member cabinet: the prime minister and the foreign minister are medical doctors, the health minister is a public health expert, and the finance minister is a former public health program manager. This was a clear advantage in their ability to understand and respond to evolving health situations. A whole-of-government response led to coordinated health response while mitigating the socioeconomic damage resulting from the collapse of tourism industry and a rising number of unemployed.

COVID-19–related public measures were implemented through the National COVID-19 Task Force led by the prime minister and supported by three regional taskforces and district taskforces.^7^ The policies at the national level were reviewed by the parliamentary committee on COVID-19. The Health Emergency Management Committee was established at the Ministry of Health. The committee was advised by the 18-member Technical Advisory Group comprising local epidemiologists, scientists, and public health experts.^18^ The taskforces at the districts were supported by the local government, civil servants, armed forces, village health workers in the community, and private individuals.

The government had made adequate planning, studied situations elsewhere, and implemented public health measures through the National COVID-19 Task force. The government took drastic actions in response to challenges that arose on the ground. With the increased risk of importing COVID-19 due to illegal smuggling of tobacco products, where the sale of tobacco was banned in the country, the government distributed tobacco products through government outlets.^19^ The government stockpiled adequate supplies of food, essential commodities, and motor vehicle fuel, and ensured adequate supply to the citizens.

There were, however, some measures of the National COVID-19 Taskforce that were criticized. Schools remained closed since March 2020, whereas the nationwide lockdown happened only in August 2020. Parents and educators criticized the loss of opportunity for schools to have remained open from March till August 2020. Schools for grades IX through XII and tertiary education colleges opened in September 2020. During the period when schools remained closed, and for students in grades preprimary through VIII, online classes were provided.

## GROSS NATIONAL HAPPINESS DOMAIN: COMMUNITY VITALITY

The WHO described the community-driven whole-of-society response to COVID-19 seen in Bhutan as exemplary.^20^ Government offices, hospitals, shops, public transport busses, and taxis adopted the Druk Trace mobile phone application that allowed quick scanning of a quick response code to register one’s presence at a location. This was later used in contact tracing of persons exposed to a case. Those without smart phones and unable to use the app were registered in a logbook maintained at these locations. All such locations provided facilities for hand sanitization or handwashing. Public transport adopted 50% carrying capacity to ensure physical distancing. Use of face mask was easily adopted by the public.

Institutions, hospitals, and government offices provided services through work from home and tele-consult. Civil society organizations such as Bhutan Kidney Foundation ensured the continued supply of immunosuppressants and dialysis services to patients, Bhutan Cancer Society ensured the continued availability of chemo- and radiation therapies, and the Royal Society of Senior Citizens ensured the delivery of medicines to older adults. The Bhutan Red Cross Society helped families conduct funerals amidst a ban on large gatherings during the lockdown period. Voluntary village health workers were trained in COVID-19 symptoms recognition, surveillance, and health advocacy measures in the villages.

Religious figures supported the government’s efforts and advocated on following the health advice on physical distancing, use of face mask, and avoidance of crowds. Religious events and community festivals were canceled or were performed behind closed doors to prevent gatherings of crowds.

An important aspect of community vitality for GNH is the donation given in charity and time spent on volunteer activities.^2^ Volunteer organizations played key roles in the COVID-19 response efforts and successful implementation of nationwide lockdown. Volunteers from the *De Suung* Guardians of Peace programme with its 15,000 members in orange uniform took up crucial roles: patrolling of borders with security agencies, monitoring of lockdown and public health safety measures, providing manpower in storing, distribution of essential goods and items to citizens, and feeding of street dogs across the country. Preventing the illegal entry of people across the borders and allowing immigration through formal routes were important areas of prevention. The entry of Indian nationals through formal points of entry followed by mandatory facility quarantine comprised 37.6% (142 of 378) of COVID-19 cases detected in the country as of November 20, 2020.^6^

The citizens and religious organizations contributed Nu 106 million (≈USD 1.4 million) to the government’s COVID-19 fund.^7^ People also offered refreshments to the volunteers and health workers, and allowed their facilities to be used by volunteers in delivering their services. Large-scale farming was adopted by farmers in the villages, and the country which otherwise was dependent on vegetable imports had adequate local productions.

Another key outcome of community participation is the implementation of a zoning system during the lockdown period and public compliance to movement regulations. The country was categorized into geographically defined zones according to the risk of COVID-19 transmission. Those in low-risk areas such as rural and high-land areas were allowed to return to normal life relatively early on, whereas high-risk areas such as Thimphu and districts along the southern borders had longer periods of lockdown.

## HOW HAS THE GNH FRAMEWORK INFLUENCED COVID-19 RESPONSE?

The constitution mandates the state to always ensure conditions for people’s happiness and well-being. The GNH spirit has manifested in government, placing people’s well-being above all and taking measures to preserve and promote health even at the cost of looming economic recession during the pandemic. The health policy response to this pandemic was influenced by compassionate leadership from the king “to safeguard the people of Bhutan” and prioritized health and health measures over economic indicators.^18^

The performance of the health system also adequately demonstrates its promptness and resilience in fighting this pandemic as well as providing routine services. Through effective public health interventions, the hospitals did not get overwhelmed with cases unlike what was predicted of low- and middle-income countries.^21^ As a result of adequate planning, preparation, simulations, and study of epidemiology and interventions elsewhere, the king and the government provided strong leadership coupled with decisive public health actions.

From March till August 2020, the country only had imported cases. Sporadic domestic cases were subsequently reported in Phuentsholing, Gelegphu, Haa, and Samdrup Jongkhar but were contained because of the rapid action of the government to test, trace, treat, and public adoption of hygiene and distancing measures. Transparent communications from the government and national address given by the king garnered public support and volunteers to deliver essential non-health services.

The COVID-19 responses were implemented by dedicated teams comprising all-Bhutanese scientists, epidemiologists, and experts. Although the country needs more health experts, this situation has demonstrated the strength of having local experts who understood the sociocultural contexts. However, there are areas where improvements can be made for the country to have adequate human resources, a robust system of surveillance, and a dedicated infectious disease hospital and infectious disease specialists. The country is dependent on supplies of personal protective equipment and consumable items from outside the country, a trend that is unreliable especially when there is a global competition and an increase in cost.^22^ The country must also invest in sustainable solutions for preparedness for future threats and challenges from pandemics, emerging and reemerging diseases.

## CONCLUSION

Learning from practices and success in COVID-19 prevention and management, the country benefitted from a home-grown development model, GNH. Bhutan has demonstrated how compassionate leadership of the King, the government advised by local experts, efficient communication and community ownership of COVID-19 response, and timely adoption of global best practices into the local sociocultural context were key to the management of this pandemic despite having limitations in infrastructure, resources, and experiences. The country however needs to learn from this experience and adopt definitive measures to overcome its health, economic, and social security challenges in an event of future health challenges and emergencies.
